# Supplementary material for: Development of a Gold Nanoparticle-Linked Immunosorbent Assay of Staphylococcal Enterotoxin B Detection with Extremely High Sensitivity by Determination of Gold Atom Content Using Graphite Furnace Atomic Absorption Spectrometry
Source: Pharmaceutics. 2023 May 13;15(5):1493. doi: 10.3390/pharmaceutics15051493 (PMC10223418; doi:10.3390/pharmaceutics15051493)
Supplement: Supplementary file 1 [file pharmaceutics-15-01493-s001.zip › pharmaceutics-2354211-supplementary.pdf]

Development of a gold nanoparticles-linked immunosorbent assay of staphylococcal enterotoxin B detection with extremely high sensitivity by determination of gold atoms content using graphite furnace atomic absorption spectrometry

Chaojun Song [1,†], Yutao Liu [2,†], Jinwei Hu [2], Yupu Zhu [2], Zhengjun Ma [2], Jiayue Xi [2], Minxuan Cui [2], Leiqi Ren [1,*] and Li Fan [2,*]

[1] School of Life Science, Northwestern Polytechnical University, Xi'an 710072, China
[2] Department of Pharmaceutical Analysis, School of Pharmacy, Air Force Medical University, Xi'an 710032, China
* Correspondence: xxfanny@fmmu.edu.cn (Li Fan), renleiqi@nwpu.edu.cn (Leiqi Ren)
† These authors contributed equally to this work.

This file includes:

(1) Supplementary figures

- Figure S1. FTIR images of AuNPs before and after modification.

(2) Supplementary tables

- Table S1. Original data for standard curve of SEB ALISA.
- Table S2. Original data for intraassay of SEB ALISA (n = 8).
- Table S3. Original data for inter-assay of SEB ALISA (n = 8).
- Table S4. Recovery data in Dilution Buffer of SEB ALISA.
- Table S5. Recoveries data of SEB detection by ALISA in various matrices.

Supplementary figures

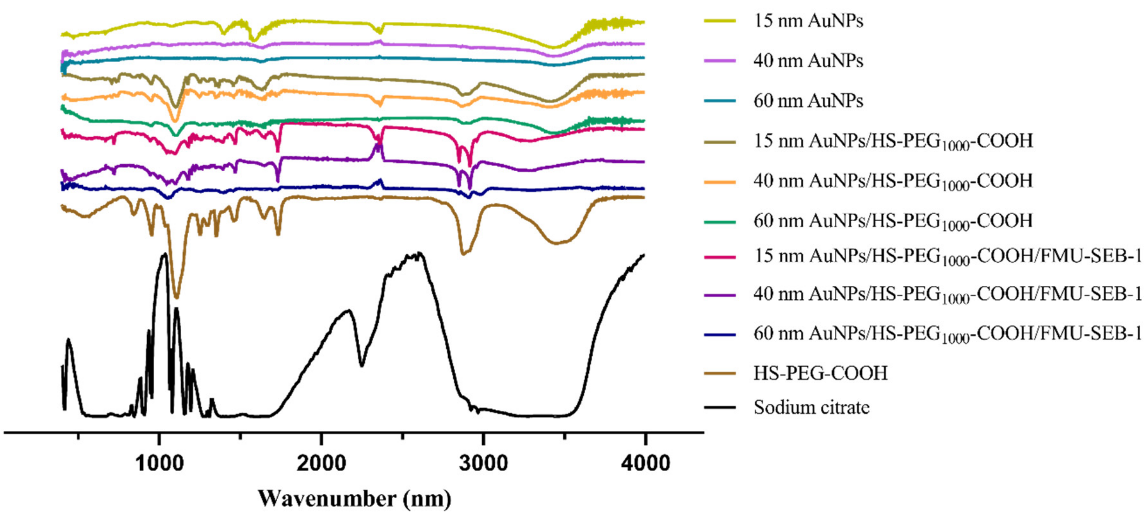

Figure S1. FTIR images of AuNPs before and after modification

Before PEG modification, the surface of gold nanoparticles was coated with sodium citrate, so that AuNPs surface showed a a broad and blunt O-H stretching vibration absorption peak at 3413 cm^{-1}. When PEG is modified on the surface, the asymmetric and symmetric stretching vibration peaks of C-H ($\nu_{asC\text{-}H}$ 2915 cm^{-1} and $\nu_{sC\text{-}H}$ 2869 cm^{-1}) will appear due to the structure of PEG, as shown in the FigS1. Finally, an amide bond is formed when the carboxyl modified gold nanoparticles are cross-linked with the antibody. In the infrared absorption spectrum, N-H stretching vibration peak ($\nu_{N\text{-}H}$) appears near 3215 cm^{-1}. This peak can be clearly distinguished from the O-H stretching vibration peak. Therefore, the above characterization indicated that the antibody was successfully labeled to the gold nanoparticles.

Supplementary tables

Table S1. Original data for standard curve of SEB ALISA.

Size (nm)	SEB concentration (pg/ mL)	Au concentration (ng/ mL)	Au concentration (ng/ mL)	Au concentration (ng/ mL)
15	5	0.278	0.356	0.394
	10	0.602	0.643	0.698
	20	1.614	1.471	1.916
	40	3.312	4.059	4.486
	80	8.047	7.429	7.033
	160	13.240	11.052	16.771
	320	29.139	21.109	25.768
	640	48.272	54.633	58.138
	1280	97.857	92.109	87.720
40	0.5	0.219	0.388	0.265
	1	0.581	0.441	0.635
	2	1.401	0.963	1.262
	4	3.041	2.413	2.727
	8	7.011	6.809	6.054
	16	14.197	11.603	17.586
	32	25.131	29.962	34.236
	64	61.115	56.418	48.622
	128	92.591	96.157	102.236
60	0.125	0.274	0.231	0.210
	0.25	0.441	0.546	0.509
	0.5	1.102	1.027	1.388
	1	2.249	2.747	2.611
	2	5.479	5.163	6.094
	4	12.493	11.761	11.128
	8	25.904	24.844	29.074
	16	53.292	45.637	46.633
	32	81.707	87.144	89.264

Table S2. Original data for intraassay of SEB ALISA (n = 8).

batch	Au concentration (ng/ mL)	SEB concentration (pg/ mL)	Au concentration (ng/ mL)	SEB concentration (pg/ mL)	Au concentration (ng/ mL)	SEB concentration (pg/ mL)
1	6.187	1.933	24.062	8.442	50.993	18.250
2	5.828	1.802	21.980	7.684	47.429	16.952
3	6.351	1.992	22.096	7.726	52.064	18.640
4	5.933	1.840	20.454	7.128	58.903	21.130
5	5.587	1.714	20.853	7.274	49.036	17.537
6	5.870	1.817	23.290	8.161	51.709	18.510
7	5.773	1.782	22.219	7.771	52.380	18.755
8	5.985	1.859	21.304	7.438	57.936	20.778

Table S3. Original data for interassay of SEB ALISA (n = 8).

batch	Au concentration (ng/ mL)	SEB concentration (pg/ mL)	Au concentration (ng/ mL)	SEB concentration (pg/ mL)	Au concentration (ng/ mL)	SEB concentration (pg/ mL)
1	6.798	2.155	21.417	7.479	62.982	22.616
2	5.593	1.716	25.356	8.913	49.291	17.630
3	5.262	1.596	20.598	7.181	55.064	19.732
4	5.834	1.804	22.361	7.823	57.732	20.704
5	5.680	1.748	28.253	9.968	47.723	17.059
6	6.612	2.087	21.957	7.676	58.490	20.980
7	6.670	2.109	23.873	8.373	50.950	18.234
8	5.085	1.531	21.360	7.458	65.765	23.629

Table S4. Recovery data in Dilution Buffer of SEB Sandwich ALISA.

	Au concentration (ng/ mL)	SEB concentration (pg/ mL)	Au concentration (ng/ mL)	SEB concentration (pg/ mL)	Au concentration (ng/ mL)	SEB concentration (pg/ mL)
	6.051	1.883	5.952	1.847	5.833	1.804
1	20.833	7.266	22.865	8.006	21.960	7.965
	51.922	18.588	51.307	18.364	53.624	19.208
	6.302	1.975	5.945	1.845	6.015	1.870
2	23.730	8.321	25.083	8.814	22.190	8.049
	52.336	18.739	53.481	19.156	50.924	18.224
	6.502	2.047	6.292	1.971	6.113	1.906
3	21.156	7.384	20.309	7.075	21.193	8.050
	58.057	20.822	61.541	22.091	60.089	21.562
	5.267	1.598	5.633	1.731	5.901	1.829
4	19.691	6.850	18.038	6.248	17.207	6.234
	45.976	16.423	43.384	15.843	49.098	17.559
	5.981	1.858	5.803	1.793	5.781	1.785
5	23.076	8.083	21.048	7.345	20.982	7.609
	52.745	18.888	50.850	18.197	52.031	18.628
	6.196	1.936	5.947	1.845	5.897	1.827
6	20.974	7.318	21.853	7.638	20.835	7.555
	58.673	21.046	57.780	20.721	57.174	20.500
	6.102	1.902	6.930	2.203	6.882	2.186
7	20.583	7.175	21.806	7.621	21.733	7.882
	51.380	18.390	52.209	18.692	52.072	18.642
	6.124	1.910	6.395	2.008	6.319	1.981
8	21.372	7.463	21.484	7.503	23.163	8.403
	48.409	17.309	50.723	18.151	48.901	17.488
	5.990	1.861	6.010	1.868	6.302	1.975
9	21.926	7.664	20.444	7.125	20.250	7.342
	48.281	17.262	49.892	17.849	45.190	16.136
	6.195	1.936	6.031	1.876	6.203	1.939
10	20.133	7.011	19.085	6.630	20.519	7.440
	49.072	17.550	50.883	18.209	44.181	16.133

Table S5. Recoveries data of SEB detection by ALISA in various matrices.

matrix	SEB concentration (pg/ mL)	Au concentration (ng/ mL)	Au concentration (ng/ mL)
Dilute buffer	0.125	0.223	0.230
	1	2.362	2.619
	4	11.710	11.923
	8	22.861	21.335
	16	44.972	43.738
	32	87.056	85.703
Cure ham	0.125	0.263	0.215
	1	2.757	2.555
	4	11.802	13.649
	8	20.461	21.070
	16	45.893	44.501
	32	91.152	95.329
Roast beef	0.125	0.242	0.203
	1	2.580	2.071
	4	11.459	9.098
	8	23.283	24.930
	16	46.369	48.874
	32	84.772	78.360
Peanut butter	0.125	0.189	0.227
	1	2.187	2.091
	4	10.135	11.310
	8	20.901	23.936
	16	41.361	37.903
	32	82.657	85.047
Human serum	0.125	0.186	0.203
	1	2.085	2.276
	4	8.943	9.762
	8	21.865	18.910
	16	39.052	37.046
	32	78.203	82.011
Ketchup	0.125	0.218	0.178
	1	2.550	2.792
	4	9.401	11.283
	8	17.606	20.027
	16	44.078	40.526
	32	80.169	76.920
Milk	0.125	0.217	0.186
	1	2.780	2.523
	4	10.387	9.721
	8	24.616	21.932

	16	40.087	42.663
	32	94.761	88.090
Orange	0.125	0.253	0.216
	1	2.101	2.501
	4	10.954	11.362
	8	20.737	17.019
	16	43.923	40.338
	32	80.274	76.641
River water	0.125	0.240	0.271
	1	2.588	2.637
	4	13.971	10.783
	8	23.642	25.173
	16	46.093	48.335
	32	94.601	88.730
Blueberry jam	0.125	0.235	0.211
	1	2.039	2.372
	4	12.703	10.829
	8	19.783	21.045
	16	39.717	41.531
	32	80.673	83.618
Soybean paste	0.125	0.232	0.267
	1	2.286	2.601
	4	11.292	14.093
	8	24.853	22.570
	16	49.083	44.234
	32	90.704	93.038
Apple juice	0.125	0.206	0.181
	1	1.932	2.349
	4	9.536	11.569
	8	20.412	23.385
	16	37.081	39.369
	32	86.701	90.819
